# Implementation of a Digital Health Technology Platform Improves Neurosurgical Resident Communication Skills

**DOI:** 10.7759/cureus.75952

**Published:** 2024-12-18

**Authors:** Harshal A Shah, Hiral V Patel, Shyle H Mehta, Daniel G Eichberg, Jung Park

**Affiliations:** 1 Department of Neurological Surgery, Donald and Barbara Zucker School of Medicine at Hofstra/Northwell, Manhasset, USA; 2 Department of Medicine, University of Maryland School of Medicine, Baltimore, USA; 3 Department of Neurosurgery, Northwell Health, Manhasset, USA

**Keywords:** communication skills, digital health, neurosurgical residency, physician-patient communication, resident education, surgical education

## Abstract

Introduction: Surgical residency lacks standardized curricula for teaching interpersonal and communication skills. We evaluated the utility of a digital health communication platform, Playback Health, that generates audiovisual recordings of patient-provider interactions as a tool for junior neurosurgical resident education.

Methods: Junior (postgraduate year (PGY)-2 and PGY-3) neurosurgical residents rated their comfort working with five attending neurosurgeons (one of whom implemented Playback Health) across 10 categories, grouped into three overarching domains encompassing knowledge base, technical skills, and interpersonal skills on a 10-point Likert scale. Ratings were performed at the beginning of the rotation at the hospital as a baseline and then every two months for a total duration of six months.

Results: At baseline, resident ratings of their comfort working with each physician did not differ significantly between the four Playback Non-Users and the Playback User. Regarding knowledge base, significant improvements arose at the four- and six-month time points regarding imaging knowledge (p = 0.045 and p = 0.003, respectively) and preoperative management (p = 0.024 and p = 0.003, respectively), and additionally for intraoperative knowledge at four months (p = 0.021) and postoperative management knowledge (p = 0.002). Among interpersonal skills, there was a significant difference with the physician who implemented Playback Health compared to Playback Non-Users as early as two months across both categories evaluated (understanding of patient concerns and expectations (p = 0.028) and understanding of patient's support structure (p = 0.005)). This difference was sustained across four- and six-month time points.

Conclusions: The implementation of a platform that generates customized audiovisual content during routine patient-provider interactions may be a valuable tool for resident education, particularly regarding interpersonal skills and understanding of patient specific concerns and support structures.

## Introduction

Surgical resident education is challenging as the acquisition of practical and conceptual skills is predicated on exposure to clinical case volumes and hands-on training in the operating room [1]. Given this challenge, the implementation of digital tools including digital training devices, virtual reality, and mobile-based applications has played a transformative role in medical education [2,3]. These tools can facilitate the understanding of operative knowledge as well as the acquisition of practical skills including technical and communication skills, with the use of media that simulates clinical practice and imparts didactic information. The ability to access digital tools on-demand makes them a valuable resource for education as they can be referenced at a time convenient for the learner and repeatedly utilized to reinforce teaching points [4-7].

A main focus of current digital tools used in surgical education is the acquisition of practical surgical skills, for example, the use of digital box trainers to augment the training of laparoscopic skills [8]. However, in addition to technical competence, the Accreditation Council for Graduate Medical Education (ACGME) emphasizes skills regarding communication, notably across two domains: patient- and family-centered communication, and interprofessional and team communication [9-11]. Despite the need for competency with communication, the acquisition of communication skills during residency is complicated by the lack of standardized communication curricula. This creates an opportunity where digital teaching tools may provide a solution.

Several reports have assessed the utility of digital resources, such as mobile applications, in surgical resident education with demonstrable improvements in resident operative and communication skills [2,3,12-14]. As technology becomes increasingly implemented in the medical setting, the role of digital resources in resident education is anticipated to grow in utilization and scope. Playback Health is a multimedia digital platform that facilitates communication between providers and patients through the use of audiovisual recordings generated by providers that are accessible to patients and their care teams [15]. The primary purpose of these recordings is for clinical care, to provide the team with a resource highlighting information the surgeon discussed with the patient. However, as content is centered around individual patients and their care, we evaluated the ability of these audiovisual recordings to serve as a resource for teaching residents in a patient-centric, case-based manner. The primary aim of this study was to determine if this mobile, video-based application can be used to enhance surgical resident teaching to improve junior resident neurosurgical knowledge, technical abilities, and interpersonal skills.

## Materials and methods

Institutional review board exemption was sought for this study. This study was conducted at a tertiary care hospital located in a large metropolitan center. Five junior neurosurgical residents (two postgraduate year (PGY)-2 and three PGY-3) evaluated their comfort in various domains working with five fellowship-trained attending academic neurosurgeons (one utilized Playback Health (Playback User) and four did not (Playback Non-Users 1-4)) in three subspecialty areas: two in cerebrovascular, one in spine, and two in cranial tumor. The faculty using Playback Health was a dual-trained cerebrovascular neurosurgeon. Scores were defined on a 10-point Likert scale as follows: 1-3, uncomfortable; 4-6, moderately comfortable; 7-9, comfortable; and 10, an ability to explain to senior residents or attendings. Evaluations were conducted by all (PGY-2 and PGY-3) residents at two time points: immediately prior to their rotation at this tertiary care hospital and after two months. Additionally, the three PGY-3 residents had longer rotations and conducted evaluations at four and six months after the start of their rotation. Evaluations were conducted in overall areas regarding knowledge base, technical skillset, and interpersonal ("soft") skills. Knowledge base was further categorized into understanding of imaging (i.e., computed tomography angiography (CTA), catheter angiography, and magnetic resonance (MR)/computed tomography (CT)), preoperative management knowledge, intraoperative knowledge (i.e., positioning, incision site, intraoperative imaging indications, and anatomy), and postoperative management knowledge. Technical skills were assessed regarding opening (i.e., soft tissue exposure, craniotomy, and dural incision), during the critical portion of the case (i.e., resection of the tumor, clipping of the aneurysm, etc.), and closure. Soft skills were assessed in subdomains regarding understanding of patient concerns and expectations, and understanding of patients' social support structure.

Descriptive statistics (i.e., means and standard errors) were calculated for each attending in each domain at the two-, four-, and six-month time points. A two-tailed Student's t-test was used to compare scores within each domain between attendings who did not use Playback Health and the attending who did. To compare changes from baseline at each time point (two months, four months, and six months), differences in scores were calculated from baselines obtained prior to the start of each resident's rotation to their respective score at two, four, and six months. At the two-month time point, changes in scores were compared between attendings for all residents. At the four- and six-month time points, changes in scores were compared between attendings for only PGY-3 residents. A paired t-test was used to compare improvements in scores from baseline to the end of the six-month rotation for all PGY-3 residents. Analyses were conducted using IBM SPSS Statistics software version 28.0.0.0 (IBM Corp., Armonk, NY) and custom scripts and functions in R (R version 4.4.1., R Foundation for Statistical Computing, Vienna, Austria). The data that supports these findings can be made available upon reasonable request.

## Results

**Figure 1:**
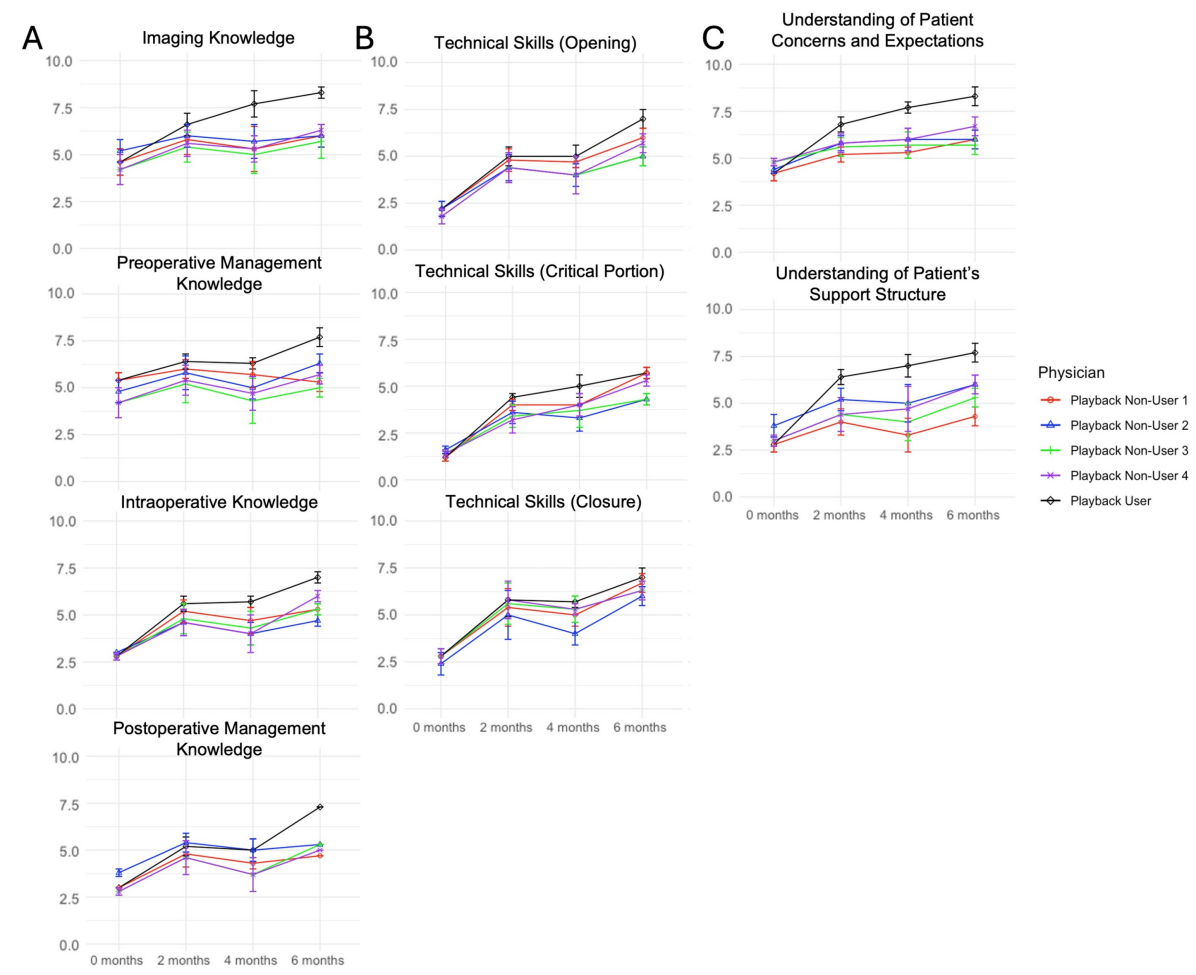
Trends over time for individual physicians The implementation of Playback Health is associated with improvements in resident comfort across multiple domains. Regarding (A) knowledge base, (B) technical skills, and (C) interpersonal skills, junior neurosurgical residents demonstrate improvements over time irrespective of Playback Health implementation, although the use of the Playback platform is associated with improvements in several knowledge base and interpersonal skill domains.

The mean resident scores for each attending by domain and attending are displayed in Table 1, and the trends over time are illustrated for individual physicians in Figure 1.

**Table 1:** Summary of average (mean) scores and standard errors for each physician in each domain at 0-, two-, four-, and six-months time points

Time	Physician	Imaging knowledge	Preoperative management knowledge	Intraoperative knowledge	Postoperative management knowledge	Technical skills (opening)	Technical skills (critical portion)	Technical skills (closure)	Understanding of patient concerns and expectations	Understanding of patient's support structure
0 months	Playback User	4.6 ± 0.7	5.4 ± 0.4	2.8 ± 0.2	3.0 ± 0.0	2.2 ± 0.4	1.2 ± 0.2	2.8 ± 0.4	4.2 ± 0.4	2.8 ± 0.4
	Playback Non-User 1	4.6 ± 0.7	5.4 ± 0.4	2.8 ± 0.2	3.0 ± 0.0	2.2 ± 0.4	1.2 ± 0.2	2.8 ± 0.4	4.2 ± 0.4	2.8 ± 0.4
	Playback Non-User 2	5.2 ± 0.6	4.8 ± 0.6	3.0 ± 0.0	3.8 ± 0.2	2.2 ± 0.4	1.6 ± 0.2	2.4 ± 0.6	4.4 ± 0.2	3.8 ± 0.6
	Playback Non-User 3	4.2 ± 0.8	4.2 ± 0.8	2.8 ± 0.2	2.8 ± 0.2	1.8 ± 0.4	1.4 ± 0.2	2.8 ± 0.4	4.8 ± 0.2	3.0 ± 0.3
	Playback Non-User 4	4.2 ± 0.8	4.2 ± 0.8	2.8 ± 0.2	2.8 ± 0.2	1.8 ± 0.4	1.4 ± 0.2	2.8 ± 0.4	4.8 ± 0.2	3.0 ± 0.3
2 months	Playback User	6.6 ± 0.6	6.4 ± 0.4	5.6 ± 0.4	5.2 ± 0.5	5.0 ± 0.5	4.4 ± 0.2	5.8 ± 0.9	6.8 ± 0.4	6.4 ± 0.4
	Playback Non-User 1	5.8 ± 0.8	6.0 ± 0.5	5.2 ± 0.6	4.8 ± 0.7	4.8 ± 0.6	4.0 ± 0.3	5.4 ± 1.0	5.2 ± 0.4	4.0 ± 0.7
	Playback Non-User 2	6.0 ± 0.6	5.8 ± 0.9	4.6 ± 0.7	5.4 ± 0.5	4.4 ± 0.7	3.6 ± 0.6	5.0 ± 1.3	5.8 ± 0.4	5.2 ± 0.6
	Playback Non-User 3	5.4 ± 0.8	5.2 ± 1.0	4.8 ± 0.8	4.6 ± 0.9	4.4 ± 0.8	3.4 ± 0.6	5.6 ± 1.1	5.6 ± 0.5	4.4 ± 0.9
	Playback Non-User 4	5.6 ± 0.7	5.4 ± 0.8	4.6 ± 0.7	4.6 ± 0.9	4.4 ± 0.8	3.2 ± 0.7	5.8 ± 1.0	5.8 ± 0.5	4.4 ± 0.9
4 months	Playback User	7.7 ± 0.7	6.3 ± 0.3	5.7 ± 0.3	5.0 ± 0.6	5.0 ± 0.6	5.0 ± 0.6	5.7 ± 0.3	7.7 ± 0.3	7.0 ± 0.6
	Playback Non-User 1	5.3 ± 1.2	5.7 ± 0.7	4.7 ± 0.7	4.3 ± 0.3	4.7 ± 0.3	4.0 ± 0.6	5.0 ± 0.6	5.3 ± 0.3	3.3 ± 0.9
	Playback Non-User 2	5.7 ± 0.9	5.0 ± 0.6	4.0 ± 0.6	5.0 ± 0.6	4.0 ± 0.6	3.3 ± 0.7	4.0 ± 0.6	6.0 ± 0.6	5.0 ± 1.0
	Playback Non-User 3	5.0 ± 1.0	4.3 ± 1.2	4.3 ± 0.9	3.7 ± 0.9	4.0 ± 1.0	3.7 ± 0.9	5.3 ± 0.7	5.7 ± 0.7	4.0 ± 1.0
	Playback Non-User 4	5.3 ± 0.7	4.7 ± 0.9	4.0 ± 1.0	3.7 ± 0.9	4.0 ± 1.0	4.0 ± 0.6	5.3 ± 0.3	6.0 ± 0.6	4.7 ± 1.2
6 months	Playback User	8.3 ± 0.3	7.7 ± 0.3	7.0 ± 0.6	7.3 ± 0.3	7.0 ± 0.6	5.7 ± 0.7	7.0 ± 0.6	8.3 ± 0.3	7.7 ± 0.3
	Playback Non-User 1	6.0 ± 0.6	5.3 ± 0.9	5.3 ± 0.9	4.7 ± 0.7	6.0 ± 0.6	5.7 ± 0.9	6.7 ± 0.3	6.0 ± 0.6	4.3 ± 1.2
	Playback Non-User 2	6.0 ± 0.6	6.3 ± 0.3	4.7 ± 0.8	5.3 ± 0.3	5.0 ± 0.6	4.3 ± 0.7	6.0 ± 0.6	6.0 ± 0.6	6.0 ± 0.0
	Playback Non-User 3	5.7 ± 0.9	5.0 ± 1.2	5.3 ± 0.9	5.3 ± 1.2	5.0 ± 1.2	4.3 ± 1.2	6.3 ± 0.3	5.7 ± 0.7	5.3 ± 0.7
	Playback Non-User 4	6.3 ± 0.3	5.7 ± 0.7	6.0 ± 0.6	5.0 ± 0.6	5.7 ± 0.7	5.3 ± 0.9	6.3 ± 0.3	6.7 ± 0.3	6.0 ± 0.6

**Figure 2:**
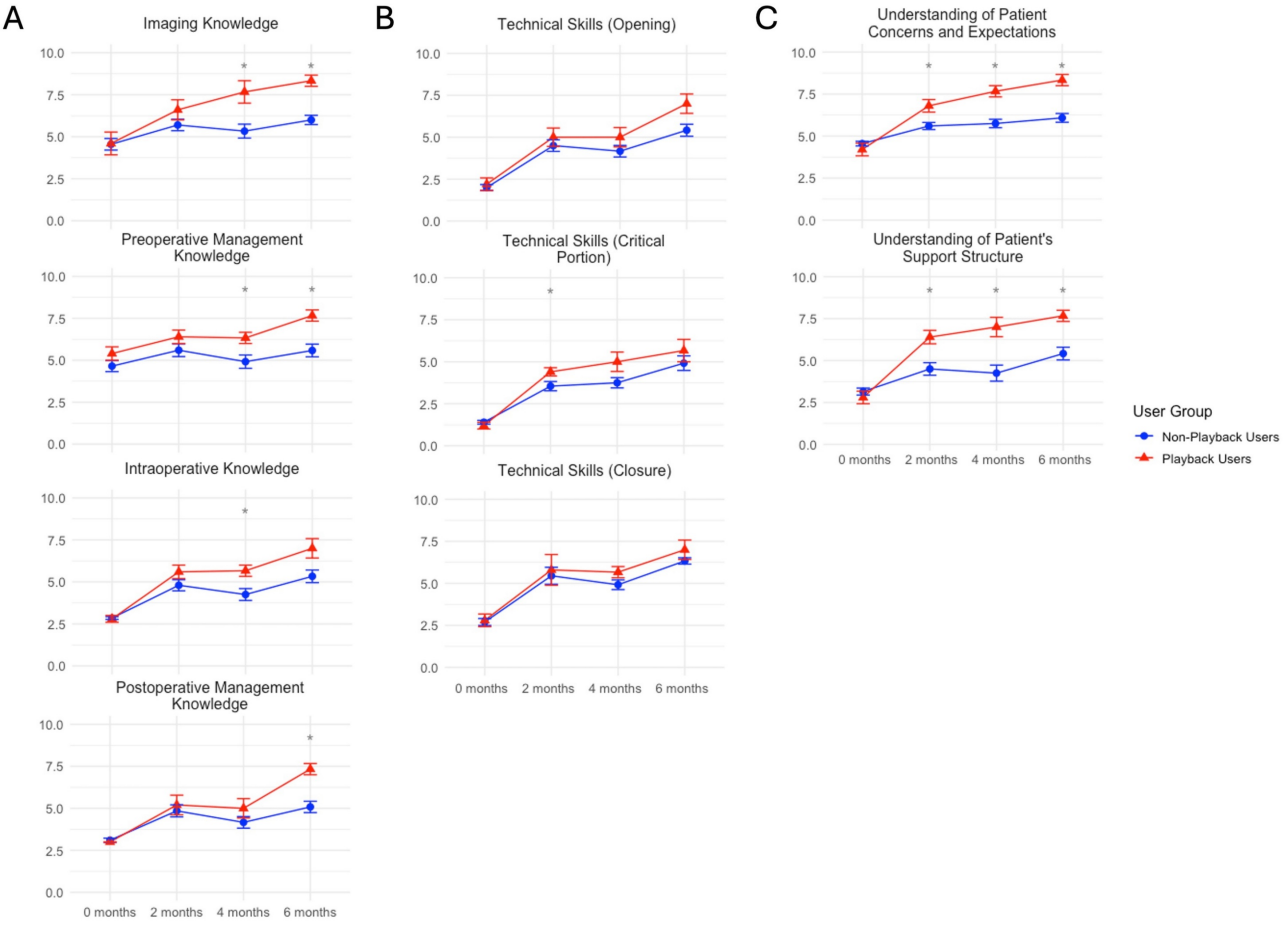
Comparison between the Playback User and the average of all non-users over time Playback Health utilization is associated with improved junior neurosurgical resident comfort across several domains. Across domains encompassing (A) knowledge base, (B) technical skills, and (C) interpersonal skills, junior neurosurgical residents demonstrate improvements over time that may be augmented by the implementation of Playback Health. "*" denotes significant differences at the p < 0.05 level.

Figure 2 illustrates the comparison between the Playback User and the average of all non-users over time. A two-tailed t-test revealed a statistically significant difference across several domains and time points. At baseline, there were no significant differences in scores of any of the domains assessed. At two months, significant differences in improvements arose in technical competency regarding the critical portion of the case (p = 0.036), and among both soft skill domains (understanding of patient concerns and expectations (p = 0.028), and patients' family support structure (p = 0.005)). At the four-month time point, significant differences were found between several knowledge domains including imaging (p = 0.045), preoperative management knowledge (p = 0.024), and intraoperative knowledge (p = 0.021). Understanding of patient concerns (p = 0.007) and support structure (p = 0.013) remained statistically significant at this time point as well. At six months, differences were significant between Playback Health users and non-users in imaging knowledge (p = 0.003), preoperative management knowledge (p = 0.003), and postoperative knowledge (p = 0.002). Intraoperative knowledge (p = 0.074) and technical skills in opening (p = 0.085) trended toward significance. Again, at the six-month time point, both soft skill domains demonstrated statistical significance (patient concerns p = 0.003; support structure p = 0.002). These results are further summarized in Table 2.

**Table 2:** Results of t-tests displayed as p values evaluating differences between resident rating scores for Playback Health user and non-user physicians at 0-, two-, four-, and six-months Significant values are denoted with an asterisk.

Time	Imaging knowledge	Preoperative management knowledge	Intraoperative knowledge	Postoperative management knowledge	Technical skills (opening)	Technical skills (critical portion)	Technical skills (closure)	Understanding of patient concerns and expectations	Understanding of patient's support structure
0 months	0.95	0.177	0.826	0.428	0.646	0.413	0.822	0.419	0.442
2 months	0.234	0.172	0.153	0.624	0.463	0.036*	0.748	0.028*	0.005*
4 months	0.045*	0.024*	0.021*	0.29	0.29	0.145	0.144	0.007*	0.013*
6 months	0.003*	0.003*	0.074	0.002*	0.085	0.4	0.369	0.003*	0.002*

Additional comparisons were performed between the physician who utilized Playback Health and another physician of the same subspecialty (cerebrovascular surgery) who did not implement Playback Health. Across knowledge base domains, technical skills, and interpersonal skills, there were no differences at baseline (0 months) between resident ratings of either physician. Among knowledge base domains, significant differences arose only at the six-month time points for imaging knowledge (p = 0.025) and postoperative management knowledge (p = 0.023). There were no differences identified in technical skills at either time point. However, among interpersonal skills, there were significant differences at the two-, four-, and six-month time points. These results are summarized in Table 3.

**Table 3:** Results of t-tests displayed as p values comparing resident rating scores for the physician who utilized Playback Health and another physician of the same subspecialty (cerebrovascular surgery) who did not utilize Playback Health across 0-, two-, four-, and six-month time points Significant differences are denoted with an asterisk.

Time	Imaging knowledge	Preoperative management knowledge	Intraoperative knowledge	Postoperative management knowledge	Technical skills (opening)	Technical skills (critical portion)	Technical skills (closure)	Understanding of patient concerns and expectations	Understanding of patient's support structure
0 months	1	1	1	1	1	1	1	1	1
2 months	0.447	0.572	0.587	0.681	0.809	0.347	0.773	0.016*	0.018*
4 months	0.165	0.422	0.251	0.374	0.643	0.288	0.374	0.008*	0.025*
6 months	0.025*	0.069	0.189	0.023*	0.288	1	0.643	0.025*	0.056

**Figure 3:**
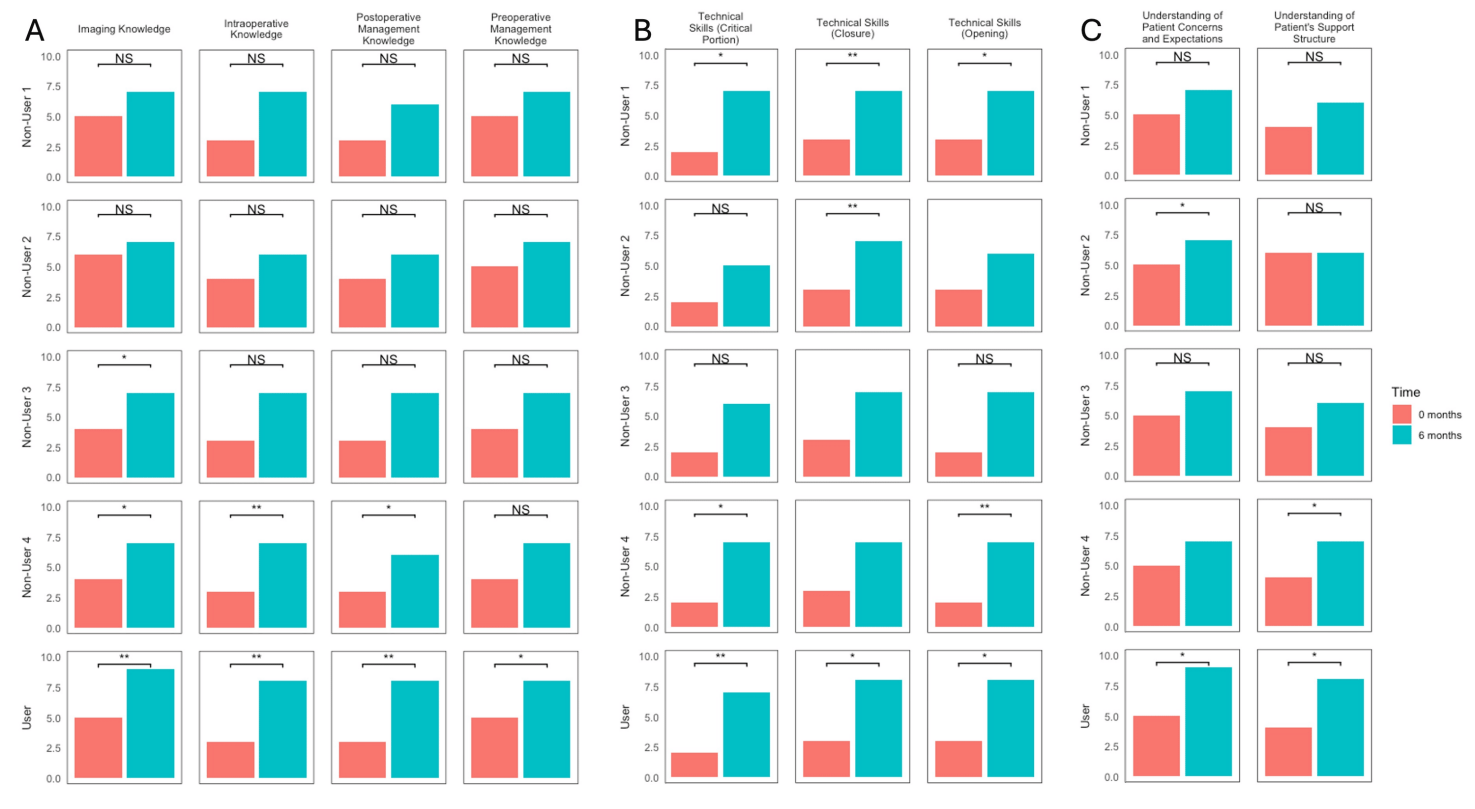
Results of paired t-tests for all PGY-3 residents Residents demonstrate significant improvements across multiple domains over time that may be augmented by Playback Health utilization. Junior neurosurgical residents demonstrated significant improvements over six months across domains encompassing (A) knowledge base, (B) technical skills, and (C) interpersonal skills. "*" denotes significance at the p < 0.05 level and "**" at the p < 0.01 level. Graphs without annotation were unable to be compared due to insufficient data variability. PGY: postgraduate year, NS: not significant

Figure 3 summarizes the results of paired t-tests for all PGY-3 residents assessing improvements from baseline scores to six months for each physician and each domain. For Playback Non-User 1, significant differences in scores from baseline were identified in technical domains regarding opening (p = 0.038), the critical portion of the case (p = 0.039), and closure (p = 0.006). For Playback Non-User 2, differences were identified regarding technical skills in closure (p = 0.006) and understanding of patient concerns and expectations (p = 0.038). Among scores for Playback Non-User 3, only scores regarding imaging knowledge demonstrated a significant change from 0 to six months (p = 0.020). Significant changes in scores for Playback Non-User 4 were observed for domains of imaging knowledge (p = 0.035), intraoperative knowledge (p = 0.009), postoperative management knowledge (p = 0.020), technical skills regarding opening (p = 0.006), technical skills during the critical portion of the case (p = 0.020), and understanding of patient support structure (p = 0.035). For the physician who implemented Playback Health, significant differences were observed for all domains as follows: imaging knowledge (p = 0.006), preoperative management knowledge (p = 0.015), intraoperative knowledge (p = 0.006), postoperative management knowledge (p = 0.006), technical skills in opening (p = 0.023), the critical portion of the case (p = 0.006), and closure (p = 0.034), and understanding of patient concerns and expectations (p = 0.023) and patient support structures (p = 0.034). Notably, for Playback Non-User 2 regarding technical skills in opening, for Playback Non-User 3 regarding technical skills in closure, and for Playback Non-User 4 in technical skills regarding closure and understanding of patient concerns domains, data lacked sufficient variability to facilitate analysis by a paired t-test.

## Discussion

Playback Health is a digital multimedia platform that allows providers to generate audiovisual informational clips during patient interactions, which can serve as a reference for patients and other providers. As these recordings are generated during routine patient interactions, such as preoperative office appointments, they do not require additional time [15]. Here, we evaluated the ability of these recordings to serve as a tool for junior resident education across several domains encompassing knowledge, technical abilities, and interpersonal skills. Our results suggest that the implementation of the Playback Health digital platform may augment the acquisition of resident skills as we observed significant improvements in scores across several categories associated with Playback Health utilization.

Across knowledge base domains, significant improvements were observed with the physician who implemented Playback Health in subdomains encompassing imaging knowledge, preoperative management, intraoperative knowledge, and postoperative management, all at the p < 0.01 level. Significant differences were not observed across Playback Non-Users 1 and 2 for any knowledge domains. For Non-User 3, improvements were observed only in imaging knowledge, and for Non-User 4, improvements were observed across all knowledge domains. Overall, scores were significantly higher for the physician who implemented Playback Health compared to the other physicians at six months (Figure 1, Figure 2). While some of these improvements may be attributed to the natural learning curve, as residents gained experience over the period of this study, the implementation of a digital teaching resource may have further augmented training. Additionally, the lack of significant difference across time points regarding knowledge base between the physician who implemented Playback Health and another physician of identical subspecialty supports that this digital resource facilitates junior resident learning. Digital resources, particularly audiovisual content, have been shown to improve learning as demonstrated by a 2018 systematic review assessing video-based education for surgical residents [4]. In a 2023 systematic review of video-based education in surgical skills education, the authors identified notable advantages of video-based education tools including improved access and greater retention, making them a valuable supplement to traditional surgical training [16]. The combination of visual and audio information through video recordings improves retention synergistically compared to either modality alone [17]. As Playback Health content is made specific to the patient with recordings generated by the patient's surgeons during the office appointment, residents may tailor their knowledge and techniques to the surgeon's and patient's specific preferences, facilitating an individualized, case-based understanding of imaging and management preferences.

Notably, our study failed to identify consistently significant differences among technical abilities regarding opening, the critical portion of the case, or closure. Although scores for several of the physicians in this study showed significant overall improvements from baseline across technical domains (Figure 3), significant differences between the physician who implemented Playback Health and those who did not were inconsistent over time (Figure 1, Table 2, Figure 2). Therefore, it is likely that these improvements were due to the natural learning curve as residents gained experience with surgical techniques, rather than the influence of implementing Playback Health. Regardless, several studies have demonstrated the ability of multimedia resources to augment the acquisition of technical skills among surgical residents. In a 2023 meta-analysis of the impact of digital tools on surgical training, digital tools were shown to significantly increase surgical skills relative to controls [18]. In a randomized, blinded study of plastic surgery residents, Kantar et al. found that the implementation of a digital cognitive simulator led to significant improvements in resident surgical knowledge, confidence in procedural aspects, and surgical performance [3]. Virtual reality trainers have also demonstrated remarkable efficacy in improving surgical resident skills across several domains [8,19,20]. The benefit of these resources in teaching residents is likely due to improved retention as a result of ordered audiovisual presentation and, in some cases, tactile stimulation, as engaging multiple sensory modalities (i.e., visual, touch, and sound cues) improves retention [21]. As the Playback Health platform lacks a robust technical simulation ability, it may not be as well suited to the acquisition of technical surgical skills relative to knowledge or soft skills.

Among the most apparent improvements in scores, however, was the difference in interpersonal skill domains observed for residents working with the physician who implemented Playback Health. From baseline, the physician who implemented Playback Health observed consistent, significant improvements in both "soft skill" domains assessed (Figure 3). This is further highlighted by the significantly higher scores for this physician at each study time point (two, four, and six months) compared to other physicians (Table 2, Figure 2) and even compared to a physician of the same subspecialty area (Table 3). The significance of these differences at the two-month time point may suggest that the implementation of this digital resource facilitates earlier acquisition of these skills. Several reports have evaluated the implementation of novel digital communication curricula for surgical residents to facilitate the acquisition of communication skills. A 2021 systematic review identified 33 studies evaluating communication skills domains studied in surgical residents, most commonly assessing themes of empathy, palliative care, error disclosure, informed consent, and shared decision-making [11]. The authors notably emphasized the lack of standardization and detailed description of communication curricula. The lack of in-depth and standardized communication curricula for surgical residents is likely because many residents acquire communication skills through observation and emulation of senior attendings; however, there remains a need for standardized communication curricula for surgical residents. In evaluating a communication curriculum for surgical residents, Newcomb et al. [14] and Newcomb et al. [22] identified that learners-determined deliberate practice can improve communication skills. This is further highlighted by the results of our study indicating that the early emphasis of communication regarding patient concerns and support structures facilitates the acquisition of resident understanding of these domains that is maintained over time.

This study has several limitations that should be considered. Although sufficient to demonstrate statistical significance, the study population was small. Furthermore, our study time frame was short, limiting the ability to extrapolate results to longer time periods. Furthermore, the residents assessed in this study were junior neurosurgical residents, and therefore, the scope of these results is limited by the homogeneity of the study subjects, types of surgery, and pathologies and may not be applicable to more senior residents or other subspecialty fields. Additionally, the lack of blinding may have introduced a source of bias to the study. Although this study serves as a proof of concept demonstrating the significance of this educational technique, longer-term, prospective studies are needed to better evaluate the effects of the implementation of this digital education tool in resident teaching.

## Conclusions

The utilization of a platform to create audiovisual didactic content, customized to individual patients, was associated with improvements in resident learning regarding knowledge base and understanding of patient-specific concerns and support structures. Critically, the use of Playback Health as a modality for education does not increase the amount of time required by the attending as it is primarily used to provide a means of communicating with the patient. Further larger studies are required to assess these results and demonstrate the utility of a patient-centric, case-based audiovisual recording format in supplementing surgical resident learning.
